# Limited Influence of Comorbidities on Length of Stay after Total Hip Arthroplasty: Experience of Enhanced Recovery after Surgery

**DOI:** 10.1111/os.12600

**Published:** 2019-12-29

**Authors:** Zi‐chuan Ding, Bing Xu, Zhi‐min Liang, Hao‐yang Wang, Ze‐yu Luo, Zong‐ke Zhou

**Affiliations:** ^1^ Department of Orthopedics, West China Hospital/West China School of Medicine Sichuan University Chengdu China; ^2^ Department of Orthopedics Chengdu Second People's Hospital Chengdu China; ^3^ Clinic Research Management Department, West China Hospital Sichuan University Chengdu China

**Keywords:** Enhanced recovery after surgery, Length of stay, predictors, Total hip arthroplasty

## Abstract

**Objectives:**

To identify predictors of length of stay (LOS) after total hip arthroplasty (THA) in an enhanced recovery after surgery (ERAS) program and evaluate the safety and cost‐efficiency of the ERAS program with reduced LOS for unselected patients in a Chinese population.

**Methods:**

A total of 311 consecutive, unselected patients undergoing primary THA at a single institution were retrospectively reviewed and divided into two groups: LOS ≤ 3 and LOS > 3 group. All patients were managed with the same ERAS protocol and went back home after discharge. Multivariate logistic regression analysis was used to determine independent risk factors for LOS > 3. Harris Hip Score at 90‐day follow‐up, 90‐day readmission rate, and hospitalization costs were compared between two groups.

**Results:**

Multivariate regression analysis identified female gender (odds ratio [OR] = 2.623), living alone (OR = 4.127), and primary osteoarthritis of hip (OR = 3.565) to be correlated with LOS > 3. Preoperative hemoglobin (HB), postoperative HB, drain use, blood transfusion, diabetes, respiratory disease, osteoporosis, number of comorbidities, and CCI score showed no significant influence on LOS after adjusting for other risk factors in the multivariate model. Harris Hip Score and readmission rate at 90‐day follow‐up showed no significant differences between two groups. Patients in LOS > 3 group had approximately 3948.6 Chinese yuan higher hospital costs.

**Conclusion:**

Female gender, living alone, and primary osteoarthritis of hip were identified as independent risk factors for prolonged LOS. The experience from our institution suggested aggressive management of comorbidities in the ERAS program can minimize the influence of comorbidities on LOS. The safety, efficiency, and costs‐saving benefits of the ERAS program with reduced LOS for unselected patients was confirmed in this study.

## Introduction

Total hip arthroplasty (THA) is the most effective and commonly used procedure for end‐stage hip disease. As the number of primary THA is expected to increase steadily, the financial burden of primary THA is projected to rise over the next 10 years^1^. The enhanced recovery after surgery (ERAS) program was pioneered by Henrik Kehlet in the 1990s to improve postoperative recovery in gastrointestinal surgery, and satisfactory results were achieved^2^. The ERAS program advocates evidence‐based interventions to decrease surgical trauma and stress response by a multimodal approach. It promotes a series of perioperative managements that focus on rapid recovery, reducing postoperative complications, early discharge, reducing costs, and improving patient satisfaction. The ERAS program has recently gained special attention in THA procedure^3^. In the ERAS program, hospital length of stay (LOS) after THA was reported to generally decline^4^, ^5^, ^6^, ^7^, ^8^, ^9^.

In the ERAS program, identifying the preoperative patient demographics related to prolonged LOS is of vital importance. It helps to optimize patient selection and predict risk stratification preoperatively. If patients undergoing THA present a predisposition for delayed discharge preoperatively, enhanced care and extended functional rehabilitation should be implemented, and the rehabilitation protocol and discharge planning for them may be changed. Determination of the perioperative risk factors for delayed discharge helps to optimize the perioperative managements for the THA patients and further improve surgical techniques. In addition, recognizing risk factors for prolonged LOS helps establish patients' expectations and provide increased patient satisfaction.

Many predictors of LOS in the ERAS program for THA procedure have been identified. Older age^5^, ^6^, ^9^, increased body mass index (BMI)^7^, female gender^5^, ^9^, living alone^6^, ^8^, and the need for blood transfusion^5^ were, among others, found to be risk factors for delayed discharge in western literatures. However, very few studies reported the perioperative predictive factors for delayed discharge in the ERAS program in a Chinese population. There are significant differences in the characteristics of THA patients between Chinese and western populations. End‐stage hip diseases have different distribution between western countries and China. High prevalence of osteonecrosis of femoral head (ONFH) and developmental dysplasia of the hip (DDH) in contrast to very low prevalence of primary hip osteoarthritis have been observed in the Chinese population^10^, ^11^. Besides, due to different races, the Chinese THA patients have much lower age and BMI than those reported abroad^6^, ^8^, ^12^. As a result, we considered that the predictors for LOS in western literatures may be nonequivalent for those of Chinese patients. Besides, there were contradictory findings with regard to the influence of comorbidities on LOS^4^, ^5^, and whether aggressive management of comorbidities in the ERAS program can limit their influence on LOS remains unclear.

It was previously demonstrated that, in the United States, shorter LOS was closely related to lower hospital costs for THA in the ERAS program^7^, ^13^. However, the relationship between LOS and hospital costs is uncertain in China due to a different medical insurance system. The perioperative managements in the ERAS program, including usage of multiple drugs, higher level of care, and improvements in surgical techniques, may lead to the increase of hospital costs. The cost‐efficiency of this value‐based program needed to be further investigated under the Chinese health‐care system. Besides, it has been reported that no associated rise in complication and readmission rates was found in the ERAS program with reduced LOS^6^, ^14^. We intended to confirm the safety and efficiency of the ERAS program with reduced LOS for unselected Chinese patients in this study.

The purpose of this study was to: (i) identify perioperative predictors of LOS after THA in a Chinese population; (ii) evaluate the cost‐efficiency of the ERAS program for THA; and (iii) evaluate the safety and efficiency of the ERAS program with reduced LOS for unselected patients undergoing THA.

## Patients and Methods

The institutional review board of our hospital approved this retrospective study and all patients provided informed consent for participation. We retrospectively reviewed 313 consecutive, unselected patients undergoing primary unilateral THA between January 2017 and June 2018 at our institution. All surgeries were performed by one experienced surgeon (ZK‐Z), performing over 600 total joint arthroplasties per year for over 10 years. The entire series of 313 patients were managed with the same ERAS protocol, which were performed by the same surgical, nursing, anesthesia, and physiotherapy team. General anesthesia and posterolateral approaches were used in the entire series of 313 patients. There were no exclusion criteria; however, two patients who were lost to follow‐up after surgery were excluded from this study. The remaining 311 patients completed the 3‐month follow‐up and were enrolled in this study.

### *ERAS Program*

**Figure 1:**
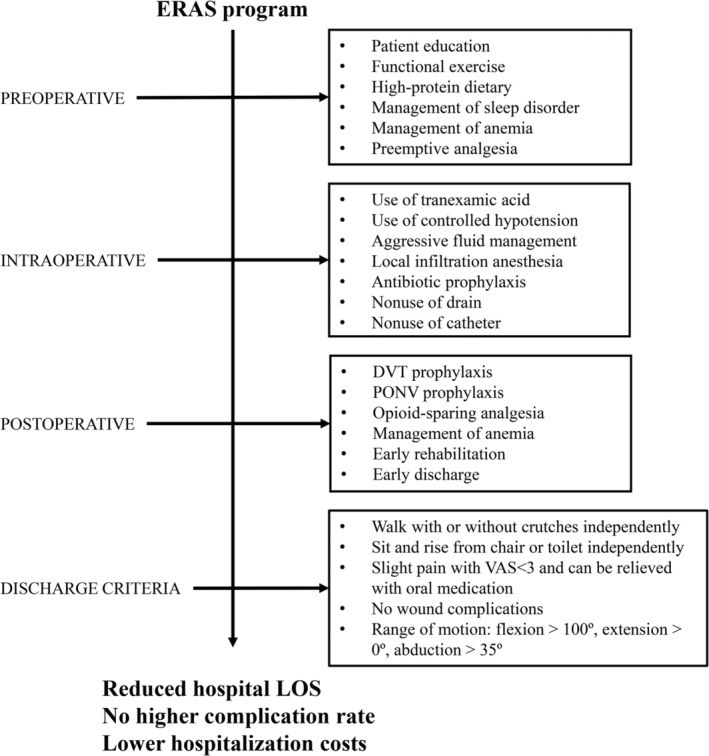
Flow chart of ERAS program. ERAS, enhanced recovery after surgery; DVT, deep vein thrombosis; LOS, length of stay; PONV, postoperative nausea and vomiting; VAS, visual analog scores.

The flow chart of the ERAS program is presented in Fig. 1. Preoperative protocols included patient education and physical therapies, such as active functional exercise to increase range of motion and strength of lower extremity. Patients were encouraged to consume a high‐protein diet to increase serum concentration of albumin perioperatively. Sleep management was utilized to treat perioperative sleep disorder, which significantly has an impact on postoperative rehabilitation and satisfaction of patients. Patients with acute insomnia received short‐term use of benzodiazepine, while the drug application for chronic insomnia was determined by neurological consultation. Aggressive fluid management was applied to limit the volume of intraoperative fluid infusion. On the day of surgery, intravenous fluid volume was controlled below 1500 ml. Perioperative multimodal pain management and blood management played an important role in our ERAS program. Perioperative multimodal pain management comprised of preoperative preemptive analgesia, intraoperative local infiltration anesthesia, and postoperative opioid‐sparing analgesia. Intraoperative blood loss was minimized through use of tranexamic acid and controlled hypotension during operation. Blood transfusions were administered for hemoglobin (HB) concentrations <70 or 70–100 g/L with symptoms. All patients received antibiotic prophylaxis and deep venous thromboembolism prophylaxis postoperatively. The functional discharge criteria were as follows: (i) able to walk independently with or without crutches; (ii) able to get in and out of bed, as well as sit and rise from chair or toilet independently; (iii) independent in transfers; (iv) slight pain with visual analogue scale (VAS) less than 3 and can be relieved with oral medication; (v) no wound complications; (vi) range of motion of the involved hip: flexion >100°, extension >0°, abduction >35°. All patients went back home after discharge and were followed up clinically and radiographically at regular intervals of 2 weeks, 1 month, 3 months, 6 months, 12 months, and annually thereafter.

### *Management of Comorbidities*

Patients with comorbidities were strictly assessed to determine if they were eligible for the surgery. Once a patient was confirmed to undergo THA, aggressive management of comorbidities was undertaken to optimize patient status, which ensures the safety of surgery and rapid rehabilitation after surgery. For example, blood pressure and glucose were controlled to be steady perioperatively. Blood pressure below 140/90 mmHg and postprandial glucose below 12.0 mmol/L were judged to be acceptable for surgery. FEV_1_/FVC was used to evaluate the pulmonary function and patients with FEV_1_/FVC over 50% were judged to be safe for THA surgery. Respiratory muscle training was used to improve pulmonary function and efficiency of cough. Erythropoietin and chalybeate were used in patients with anemia both pre‐ and post‐operatively, and the preoperative HB should be over 100 g/L. Patients diagnosed with coronary heart disease or with angina should undergo myocardial perfusion imaging test, and the myocardial blood flow over 90% was judged to be safe for THA surgery.

## Data Collection

### *Postoperative Length of Stay*

Postoperative LOS was defined as the number of nights stayed in hospital after surgery. The target postoperative LOS in our institution was 3 days^15^, thus we divided the patients into two groups: LOS ≤ 3 and LOS > 3 groups.

### *Demographics and Surgical Factors*

Patient demographics of age, gender, BMI, preoperative Harris Hip Score (HHS), operation history of involved hip and diagnosis were collected from clinical records. Living alone was defined as living without any partner in the last 6 months. Surgical factors, including use of catheters, use of drains, and blood transfusions, were collected from operation records and clinical records. Intraoperative estimated blood loss (EBL) was calculated according to Gross^16^: EBL(L) = EBV × (Hct_pre_−Hct_po_)/Hct_mean_. Hct_pre_ and Hct_po_ was the preoperative and postoperative hematocrit level, respectively. Hct_mean_ was the average of Hct_pre_ and Hct_po_. Estimated blood volume (EBV) was calculated according to Nadler *et al*.^17^: EBV_woman_ (L) = height (m)^3^ × 0.356 + body weight (kg) × 0.033 + 0.183; EBV_man_ (L) = height (m)^3^ × 0.367 + body weight (kg) × 0.032 + 0.604.

### *Comorbidities*

Existing comorbidities which were diagnosed prior to admission were documented in the clinical records in detail. Comorbidities diagnosed perioperatively were also recorded. American Society of Anesthesiologists (ASA) score, Charlson Comorbidity Index (CCI), and number of comorbidities were recorded to assess the general health status of patients and the presence and severity of comorbidities^18^, ^19^, ^20^. Laboratory evaluations before surgery and on the first postoperative day, including HB and albumin, were collected.

### *Follow‐up Function, Readmission, and Hospitalization Costs*

The hip function at 90‐day (3‐month) follow‐up was evaluated by one observer, who had not participated in the operation and clinical care of these patients, using HHS (0–100, best score: 100). At 90‐day follow‐up, patients were asked specifically if they had readmitted into hospital after discharge. Information about readmission into our institution was routinely documented and collected. Complications causing readmission, treatments and outcomes were collected in detail. Patients readmitted into other institutions were asked to provide clinical records. Hospitalization costs for each patient, except that for prostheses, were recorded from our hospital information system.

### *Statistical Analysis*

Descriptive and univariate analyses were initially conducted to examine the relationship between LOS and variables. Continuous variables, including age, BMI, HB, albumin, estimated blood loss, HHS, and hospital costs, were presented as the mean ± standard deviation. Categorical variables, including gender, living situation, operation history, diagnosis, catheter use, drain use, blood transfusion, comorbidities, CCI score, ASA score, and complications, were presented as numbers with percentages. Independent *t* test was used to compare continuous variables and Pearson chi‐square test was used to analyze categorical variables between the two groups. Multivariate logistic regression analysis was used to determine independent risk factors for prolonged LOS in variables which showed *P* < 0.1 in *t* test and Pearson chi‐square test. The level of significance was defined as *P* < 0.05 and all statistical analysis was performed with SPSS v22.0 (IBM, Armonk, NY).

## Results

### *Postoperative Length of Stay*

Of the 311 enrolled patients, 196 patients (63.02%) discharged on post‐operation day 1–3 (LOS ≤ 3 group) and 115 patients (36.98%) discharged on post‐operation day 4 or later (LOS > 3 group). The mean postoperative LOS was 3.69 ± 1.96 days.

### *General Results*

The average age was 54.98 ± 14.51 years (range, 19–86 years) and the average BMI was 23.32 ± 3.68 kg/m^2^ (range, 14.26–33.30 kg/m^2^) of all 311 patients. No significant differences were observed in age and BMI between the two groups (Table 1). There were 165 female patients (53.05%, mean age: 56.29 ± 15.20 years) and 146 male patients (46.95%, mean age: 53.51 ± 13.56 years) in the total cohort. LOS > 3 group had significantly higher proportion of female patients than LOS ≤ 3 group (69.2% *vs* 45.9%, *P* = 0.001). LOS > 3 group had significantly higher proportion of patients who lived alone than LOS ≤ 3 group (18.3% *vs* 5.1%, *P* < 0.001). Diagnosis showed significant difference between two groups (Table 1). Patients in LOS > 3 group showed significantly lower preoperative and postoperative HB than patients in LOS ≤ 3 group. No significant differences were observed in preoperative HHS, operation history of involved hip, preoperative and postoperative albumin between the two groups.

**Table 1:** Demographics and surgical factors

Baseline data	LOS ≤ 3 (n = 196)	LOS > 3 (n = 115)	*P* value
Demographic characteristics			
Age (years)	55.7 ± 13.4	53.6 ± 16.2	0.243
Gender (female)*	90 (45.9%)	75 (65.2%)	0.001
BMI (kg/m^2^)	23.1 ± 3.5	23.7 ± 4.0	0.210
Living situation (living alone)*	10 (5.1%)	21 (18.3%)	<0.001
Preoperative HHS	55.6 ± 10.6	53.9 ± 13.4	0.251
Operation history of involved hip	10 (5.1%)	10 (8.7%)	0.212
Diagnosis*			0.020
ONFH	90 (45.9%)	35 (33.4%)	‐
DDH	69 (35.2%)	36 (31.3%)	‐
Primary osteoarthritis	13 (6.6%)	16 (13.9%)	‐
Suppurative arthritis	7 (3.6%)	12 (10.4%)	‐
Rheumatoid arthritis	4 (2.0%)	4 (3.5%)	‐
Fracture of femoral neck	4 (2.0%)	5 (4.3%)	‐
Legg‐Calve‐Perthes disease	6 (3.1%)	5 (4.3%)	‐
Ankylosing spondylitis	3 (1.5%)	2 (1.7%)	‐
Laboratory values			
Preoperative HB*	135.8 ± 15.9	130.1 ± 16.2	0.002
Postoperative HB*	115.4 ± 15.2	108.3 ± 15.6	<0.001
Preoperative albumin	44.9 ± 4.3	44.4 ± 4.4	0.346
Postoperative albumin	36.7 ± 3.8	36.2 ± 3.6	0.258
Operative variables			
Catheter use	13 (6.6%)	8 (7.0%)	0.913
Drain use*	6 (3.1%)	12 (10.4%)	0.007
Estimated blood loss (mL)	662.8 ± 471.0	672.5 ± 448.0	0.859
Blood transfusion*	1 (0.5%)	4 (3.5%)	0.064

BMI, body mass index; DDH, developmental dysplasia of hip; HB, hemoglobin; HHS, Harris Hip Score; ONFH, osteonecrosis of femoral head.

* Variables with *P* value < 0.1 were further included in the multivariate logistic regression model.

In regard to surgical factors, drains were more often used in LOS > 3 group than in LOS ≤ 3 group (3.1% *vs* 10.4%, *P* = 0.007). No significant differences were observed in catheter use, estimated blood loss, and blood transfusion between two groups. Variables with *P* < 0.1, including gender, living situation, diagnosis, preoperative HB, postoperative HB, drain use, and blood transfusion, were then examined by multivariate logistic regression analysis to determine independent risk factors for LOS > 3.

### *Comorbidities*

Patients with comorbidities accounted for 44.7% of the cohort, with 24.4% presenting one comorbidity, 15.4% presenting two comorbidities, and 4.8% presenting three or more comorbidities (Table 2). No single comorbidity showed significant difference between the two groups. Patients in LOS ≤ 3 group had less comorbidities and lower CCI score than patients in LOS > 3 group (*P* = 0.028 and 0.030, respectively). Diabetes, respiratory disease, osteoporosis, number of comorbidities, and CCI score, showing *P* < 0.1 in univariate analysis, were then examined by multivariate logistic analysis to determine independent risk factors for LOS > 3.

**Table 2:** Comorbidities

Comorbidities	LOS ≤ 3 (n = 196)	LOS > 3 (n = 115)	*P* value
Diseases			
Hypertension	44 (22.4%)	26 (22.6%)	0.974
Atherosclerosis	15 (7.7%)	7 (6.1%)	0.603
Diabetes*	7 (3.6%)	10 (8.7%)	0.055
Respiratory disease*	5 (2.5%)	8 (7.0%)	0.079
Renal disease	9 (4.6%)	9 (7.8%)	0.238
Liver disease	9 (4.6%)	10 (7.0%)	0.145
Autoimmune disease	8 (4.1%)	7 (6.1%)	0.426
Osteoporosis*	18 (9.2%)	19 (16.5%)	0.054
Tumor	4 (2.0%)	2 (1.7%)	0.852
Number of comorbidities*			0.028
0	112 (57.1%)	60 (52.2%)	‐
1	54 (27.6%)	22 (19.1%)	‐
2	22 (11.2%)	26 (22.6%)	‐
≥3	8 (4.1%)	7 (6.1%)	‐
CCI score*			0.030
0	42 (21.4%)	39 (33.9%)	‐
1	57 (29.1%)	20 (17.3%)	‐
2	43 (21.9%)	24 (20.9%)	‐
3	36 (18.4%)	23 (20.0%)	‐
4	9 (4.6%)	8 (7.0%)	‐
≥5	9 (4.6%)	1 (0.9%)	‐
ASA score			0.180
1	50 (25.5%)	35 (30.4%)	‐
2	108 (55.1%)	51 (44.3%)	‐
≥3	38 (19.4%)	29 (25.2%)	‐

ASA, American Society of Anesthesiologists; CCI, Charlson comorbidity index.

* Variables with *P* value < 0.1 were further included in the multivariate logistic regression model.

### *Multivariate Logistic Regression Analysis*

Gender, living situation, diagnosis, preoperative HB, postoperative HB, drain use, blood transfusion, diabetes, respiratory disease, osteoporosis, number of comorbidities, and CCI score were included in multivariate logistic analysis model (Table 3).

**Table 3:** Multivariate logistic regression analysis identifying variables related to LOS > 3

Variable	Odds ratio	95% confidence interval	*P* value
Female*	2.623	1.330–5.173	0.005
Living alone*	4.127	1.573–10.828	0.004
Diagnosis			0.027
ONFH (reference)	1	‐	‐
DDH	0.861	0.423–1.745	0.681
Primary osteoarthritis*	3.565	1.986–6.672	0.033
Suppurative arthritis	2.453	0.738–8.152	0.143
Rheumatoid arthritis	1.161	0.195–6.927	0.870
Fracture of femoral neck	2.224	0.416–11.881	0.350
Legg‐Calve‐Perthes disease	2.225	0.416–11.881	0.282
Ankylosing spondylitis	1.431	0.184–11.110	0.732
Preoperative HB	1.000	0.973–1.027	0.974
Postoperative HB	0.982	0.957–1.008	0.168
Drain use	2.106	0.655–6.772	0.212
Blood transfusion	4.620	0.164–130.068	0.369
Diabetes	2.196	0.640–7.540	0.211
Respiratory disease	3.215	0.766–13.487	0.110
Osteoporosis	1.435	0.517–3.988	0.488
Number of comorbidities			0.412
0	1	‐	‐
1	0.628	0.307–1.284	0.202
2	1.187	0.472–2.980	0.716
≥3	0.622	0.119–3.236	0.572
CCI score			0.148
0	1	‐	‐
1	0.381	0.176–0.828	0.015
2	0.501	0.230–1.100	0.085
3	0.593	0.225–1.289	0.165
4	0.935	0.269–3.246	0.915
≥5	0.255	0.040–0.605	0.145

CCI, Charlson comorbidity index; DDH, developmental dysplasia of hip; HB, hemoglobin; ONFH, osteonecrosis of femoral head.

* Showed statistical significance in multivariate model.

In multivariate logistic analysis model, female gender (odds ratio [OR] = 2.623, 95% confidence interval [CI] = 1.330–5.173, *P* = 0.005), living alone (OR = 4.127, 95% CI = 1.573–10.828, *P* = 0.004), and primary osteoarthritis of hip (OR = 3.565, 95% CI = 1.986–6.672, *P* = 0.033) were identified as independent risk factors for LOS > 3. Primary osteoarthritis of hip showed significantly higher risk for prolonged LOS when compared with ONFH, while other primary diagnoses didn't show significant influence on LOS when compared with ONFH. Preoperative HB, postoperative HB, drain use, blood transfusion, diabetes, respiratory disease, osteoporosis, number of comorbidities, and CCI score were not identified as independent risk factors for prolonged LOS in this multivariate model after adjusting for other factors, and were determined to be confounding variables.

### *Follow‐up Function, Readmission, and Hospitalization Costs*

The mean HHS at 90‐day follow‐up was 94.6 ± 4.5 and 93.8 ± 5.5 in LOS ≤ 3 and LOS > 3 group (*P* = 0.195), respectively (Table 4).

**Table 4:** 90‐day complications and hospital costs

Parameter	LOS ≤ 3 (n = 196)	LOS > 3 (n = 115)	*P* value
Follow‐up HHS	94.6 ± 4.5	93.8 ± 5.5	0.195
Readmission	5 (2.6%)	8 (7.0%)	0.079
Dislocation	4 (2.0%)	4 (3.5%)	‐
Periprosthetic fracture	1 (0.5%)	0	‐
Pneumonia	0	1 (0.9%)	‐
Fasciitis of thigh	0	1 (0.9%)	‐
Scrotal hematoma	0	1 (0.9%)	‐
Poor nutrition	0	1 (0.9%)	‐
Hospital costs (Chinese yuan)*	22669.3 ± 3043.2	26617.9 ± 4605.1	<0.001

HHS, Harris hip score.

* Showing significant difference.

The 90‐day readmission rates were 2.6% (5/196) in LOS ≤ 3 group and 7.0% (8/115) in LOS > 3 group, with no significant difference between two groups (*P* = 0.079). In LOS ≤ 3 group, four cases of postoperative dislocation occurred and were all successfully treated with closed or open reduction, and one of them was followed with abduction bracing for 3 months. One periprosthetic femoral fracture occurred with major trauma and was revised to a cementless long stem. In LOS > 3 group, the most common complication was dislocation (four cases), followed by pneumonia (one case), fasciitis of thigh (one case), scrotal hematoma (one case), and poor nutrition (one case). Dislocations were all successfully managed by non‐operative treatment in LOS > 3 group. Scrotal hematoma occurred secondary to adductor tenotomy in a patient with severe restricted abduction of the hip and was successfully treated by open debridement.

The mean costs were 22669.3 ± 3043.2 Chinese yuan for LOS ≤ 3 group and 26617.9 ± 4605.1 yuan for LOS > 3 group (*P* < 0.001), suggesting that longer hospital LOS was associated with higher costs (Table 4).

## Discussion

LOS after primary THA was reported to generally decline in the ERAS program without associated rise in complication and readmission rates^4^, ^5^, ^6^, ^7^, ^8^, ^9^, ^14^. Identifying predictors of prolonged LOS in ERAS programs helps to optimize patient selection, predict risk stratification, improve perioperative management, and improve patients' satisfaction. Recent studies have reported multiple risk predictors for prolonged LOS, which were defined as over 1–4 days hospital stay in different studies^4^, ^5^, ^6^, ^7^, ^8^, ^9^, ^14^. However, few previous studies reported preoperative and perioperative predictors of LOS after THA in a Chinese population. Yang *et al*. reported that 2‐day discharge after THA was feasible and safe in a clinical trial of 126 selected Chinese patients. Age, operative time, and blood loss were identified as predictors for prolonged LOS^21^. However, selected patients with strict inclusion criteria and small sample size in this study may be not a good representation of all patients in real‐world clinical practice. In this current study, we analyzed data from 311 consecutive, unselected patients in a single medical team under the same ERAS protocol. Our study demonstrated that female gender, living alone, and primary hip osteoarthritis were independent predictors for prolonged LOS. Despite a different medical insurance system, the cost‐efficiency of THA was also improved by applying the ERAS program and early discharge. Besides, we confirmed the safety and efficiency of the ERAS program with reduced LOS for unselected Chinese patients undergoing THA procedure.

We found that female gender was 2.62 times more likely to have LOS > 3, which is in accordance with previous findings showing that females had a higher chance of increased hospital stay^7^, ^8^, ^9^, ^22^, ^23^. Studies attributed this to be due to the later stage of disease and worse functional status female patients had before surgery^24^, ^25^. However, we didn't find any difference in preoperative HHS between female and male (54.96 ± 11.09 *vs* 54.93 ± 12.25, *P* = 0.196). We speculated that muscle weakness and decreased soft‐tissue tension which were common in female patients may lead to slower postoperative rehabilitation and further result in prolonged LOS.

Recent studies have identified living alone as a predictor for delayed discharge^5^, ^6^, ^8^, ^26^. Our results confirmed this association. This may be explained by logistical issues as patients who live alone are unwilling to discharge early because no one will care for them after discharge. Patients living alone may be improper and ineligible for early discharge and the importance of preoperative education for them should be noted.

Age was not found to be an independent factor to predict LOS in this study, possibly because the mean age in this study was much lower than previously reported. The mean age of this cohort was 54.8 years, in contrast to previously reported 64.7–71.0 years in western countries^5^, ^6^, ^7^, ^8^, ^26^, ^27^. However, data of 7477 patients from 26 university teaching hospitals in China suggested that the mean age of Chinese THA patients was 57.6 ± 18.2 years^12^. Besides, Chan *et al*. reported the mean age of 419 Chinese patients underwent primary THA was 57.6 ± 16.6 years^28^. As a result, we believe the patients in our cohort were a good representative of the age range of the Chinese THA population. High prevalence of ONFH and DDH, different economic status, lower life expectancy, and different perspectives on medicine may explain the lower age in patients undergoing THA in the Chinese population.

With regard to the influence of BMI on LOS, there was conflicting evidence in the literature. Some studies reported a positive correlation between BMI and LOS^7^, ^22^, ^29^, ^30^ while others suggested no association between higher BMI and longer LOS^4^, ^5^, ^8^, ^9^, ^23^. Maradit Kremers *et al*. from Mayo Clinic demonstrated a J‐shaped relationship between BMI and LOS. Patients with BMI of 25–35 had lowest LOS and BMI over 30 was associated with longer LOS^30^. The mean BMI was 23.32 and only 17 patients (5.5%) had BMI over 30 in our study, which was consistent with previously reported lower BMI in Chinese people^31^, thus it is reasonable that BMI was not determined as a predictor for LOS > 3 in this study.

High prevalence of ONFH and DDH in contrast to very low prevalence of primary hip osteoarthritis have been observed in Chinese population^10^, ^11^. Patients included in previous studies were mostly diagnosed as having primary hip osteoarthritis^23^, ^32^, and different diagnoses have rarely been proved as predictors for LOS. Huang *et al*. found different diagnoses, including ONFH, primary osteoarthritis, and rheumatoid arthritis, had no significant effects on LOS in an Asian population^33^. Contrary to previous studies, we found primary osteoarthritis had higher risk for LOS > 3 with ONFH being a reference, while DDH showed no influence on LOS. This may be explained by slow progression and long course of primary OA, which lead to impairment in lower limb strength, restriction of range of motion, and slower postoperative recovery.

We combined number of comorbidities, ASA score, and CCI score to evaluate the presence and severity of comorbidities. All of these tools have been proven to be valid and reliable for measuring comorbidities in patients undergoing THA^18^, ^19^, ^20^. The influence of comorbidities on LOS was shown to be limited in our multivariate regression model, which differed from the conclusion drawn from most previous studies^34^, ^35^. We speculated that all of the following reasons may explain the finding in our study. The perioperative management of comorbidities was accurate and aggressive in our unit and we also tried to keep the condition of comorbidities stable by utilizing minimally invasive surgery technique to decrease surgical stress. As a result, the influence of comorbidities on LOS was minimized. Besides, only 44.2% of the patients presented with comorbidities and only 4.7% presented with three or more comorbidities. The proportion of patients with comorbidities was smaller than previously reported^22^, ^27^, ^36^. Le Mar and Whitehead reported that over 63% patients had at least one comorbidity in their cohort^27^ and it was reported that over 65% of patients had at least five comorbidities in another study^22^. Furthermore, the mean age of this cohort was 54.8 years while it has been reported that the mean age of patients undergoing THA ranged from 64.7 to 71.0 years in western studies^5^, ^6^, ^7^, ^8^, ^26^, ^27^. Given the fact that the patients in our cohort were notably younger than previously reported, we can speculate that patients in our study had better potential compensatory capacity for comorbidities and better general health status. As a result, comorbidities were proven to have limited influence on LOS in our study.

The safety and efficiency of the ERAS program with reduced LOS for unselected patients undergoing THA were confirmed in this study^14^, ^37^. The mean HHS at 90‐day follow‐up showed no significant difference between LOS ≤ 3 and LOS > 3 group. In addition, no difference was observed in 90‐day readmission rate between the two groups. Early discharge had no side effects on functional recovery, complication, and readmission rates. Patients in LOS > 3 group had approximately 3948.6 Chinese yuan higher hospital costs when compared to patients with LOS ≤ 3. The cost‐efficiency of the ERAS program was validated in this study; it is effective to reduce hospital costs through promotion of early discharge under the Chinese health care system.

There are some limitations in this study. First, the number of patients included in this study was relatively small. However, 311 consecutive, unselected patients in a single medical team treated by the same experienced surgeon gave our conclusion considerable power. Unlike data from several institutions or national registers, the ERAS protocols and surgical techniques in our study were consistent. Second, LOS > 3 was regarded as delayed discharge in this study. Several recent studies in the United States demonstrated the efficiency of discharge on post‐operation day one or on the day of surgery^7^, ^23^. However, it may not be applicable in the Chinese population because of a different perspective on medicine, medical care policy, and socioeconomic issues. Third, some variables that may have effects on LOS were unknown and not included in our analysis, the most important of which was patients' expectancy to discharge. We tried to minimize the influence of this through preoperative patient education of our early discharge protocol. Fourth, a recent study found that count of medication was an effective way to evaluate the severity of comorbidities and predict LOS after THA^38^. However, the count of medication was not recorded in the past in our institution and the retrospective nature of this study limited its application.

### *Conclusion*

Female gender, living alone, and primary osteoarthritis of hip were identified as independent risk factors for LOS > 3. Comorbidities, number of comorbidities, and CCI score showed no significant influence on LOS after adjusting for other risk factors in multivariate regression model. Despite younger age and smaller proportion of patients with comorbidities in our cohort, the experience from our institution suggested aggressive management of comorbidities in the ERAS program can minimize the influence of comorbidities on LOS. It is safe for THA patients with LOS less than 3 days to go home in a Chinese population. The cost‐saving benefits of the ERAS program with reduced LOS for THA were confirmed in this study.
